# Dysnatremia and Endocrine Alterations in Hospitalized COVID-19 Patients: Markers of Systemic Stress Rather than Independent Predictors of Outcomes

**DOI:** 10.3390/biomedicines14081854

**Published:** 2026-08-18

**Authors:** Mihaela Zlosa, Luka Švitek, Barbara Grubišić, Nika Vlahović Vlašić, Petra Smajić, Dario Sabadi, Ines Bilić-Ćurčić, Tara Rolić, Tomislav Kizivat

**Affiliations:** 1 Clinic for Infectious Diseases, University Hospital Centre Osijek, 4 Josip Huttler Street, HR-31000 Osijek, Croatia; mihaela.zlosa@kbco.hr (M.Z.);; 2 Department for Microbiology and Infectious Diseases, Faculty of Medicine Osijek, J. J. Strossmayer University of Osijek, 4 Josip Huttler Street, HR-31000 Osijek, Croatia; 3 Faculty of Dental Medicine and Health Osijek, J. J. Strossmayer University of Osijek, 21 Crkvena Street, HR-31000 Osijek, Croatia; 4 Department of Endocrinology and Metabolism Disorders, Clinic for Internal Medicine, University Hospital Centre Osijek, 4 Josip Huttler Street, HR-31000 Osijek, Croatia; 5 Department of Pharmacology, Faculty of Medicine Osijek, J. J. Strossmayer University of Osijek, 4 Josip Huttler Street, HR-31000 Osijek, Croatia; 6 Institute of Clinical Laboratory Diagnostics, University Hospital Centre Osijek, 4 Josip Huttler Street, HR-31000 Osijek, Croatia; tararolic@gmail.com; 7 Clinical Institute for Nuclear Medicine and Radiation Protection, University Hospital Centre Osijek, 4 Josip Huttler Street, HR-31000 Osijek, Croatia; 8 Department for Nuclear Medicine, Faculty of Medicine, J. J. Strossmayer University of Osijek, 4 Josip Huttler Street, HR-31000 Osijek, Croatia

**Keywords:** COVID-19, SARS-CoV-2, hyponatremia, endocrine system diseases, stress, physiological, biomarkers

## Abstract

**Background:** COVID-19 is a multisystem disorder associated with inflammatory, metabolic, electrolyte, and endocrine disturbances. However, the clinical relevance of dysnatremia and acute hormonal alterations remains uncertain, particularly when assessed alongside established clinical risk factors. **Methods:** This prospective cohort study included 252 adults hospitalized with confirmed COVID-19. Clinical, laboratory, hormonal, and metabolic parameters were assessed at admission, and their associations with disease severity, intensive care unit (ICU) admission, and in-hospital mortality were analyzed. **Results:** The median age was 73 years, 50.4% of patients were female, and severe or critical disease was present in 81.7%. Dysnatremia and/or endocrine alterations were detected in 55.2% of patients, including hyponatremia in 16.7%, while thyroid-function alterations were observed in 40.9%. Dysnatremia was associated with higher glucose levels and inflammatory-cell changes. Several hormonal and metabolic parameters showed statistically significant univariate associations with adverse outcomes; however, their discriminative performance was weak, with AUC values below clinically meaningful thresholds. After adjustment for major clinical confounders, dysnatremia and endocrine alterations were not independent predictors of disease severity, ICU admission, or mortality. **Conclusions:** These findings suggest that electrolyte and endocrine abnormalities in hospitalized COVID-19 patients primarily reflect systemic stress physiology and acute disease burden rather than functioning as independent prognostic biomarkers.

## 1. Introduction

Regardless of being initially classified as a respiratory illness, COVID-19 has since become widely accepted as a complex multisystem disorder with substantial rates of morbidity and death, especially within the inpatient setting [1]. Although the global burden of COVID-19 has substantially decreased since the beginning of the pandemic, hospitalized patients continue to experience severe complications, including respiratory failure, multiorgan dysfunction, intensive care unit admission, and death, highlighting the ongoing need for reliable prognostic biomarkers [2,3]. Multiple studies have shown that COVID-19 severity and the risk of hospitalization are strongly associated with older age, comorbidities, and elevated inflammatory parameters [4,5,6]. Despite advances in vaccination have significantly improved COVID-19 outcomes, severe disease requiring hospitalization remains an important clinical challenge. Consequently, the identification of readily available prognostic biomarkers continues to be a priority in the management of hospitalized patients [7]. In clinical practice, COVID-19 is categorized into mild, moderate, severe, and critical groups. As waves of disease due to variants have emerged, the proportion of cases that have been mild to moderate have varied throughout the pandemic. Although most SARS-CoV-2 infections follow a mild clinical course, hospitalized patients represent a population with a substantially higher risk of severe and critical disease [8]. A high incidence of comorbidities and the occurrence of various metabolic and biochemical alterations compound the disease severity. Studies indicate that the presence of diabetes mellitus independently raises the risk for oxygen support, mechanical ventilation, and mortality in patients suffering from COVID-19. Moreover, arterial hypertension, cardiac failure, and atrial fibrillation are associated with increased disease severity and unfavorable clinical outcomes [9,10]. Among the systemic manifestations of COVID-19, electrolyte and hormonal alterations have received emphasis because they may be indicative of the intensity of inflammatory, metabolic, and neuroendocrine stress. However, their independent prognostic value remains inconclusive and should not be assumed by unadjusted associations alone. Alterations in thyroid function, cortisol secretion, glucose metabolism, and insulin resistance have frequently been described in hospitalized patients with COVID-19, revealing activation of neuroendocrine and metabolic stress pathways [11,12,13,14,15]. In addition, vitamin D deficiency and altered insulin-like growth factor 1 (IGF-1) have been reported, suggesting wider disruption of endocrine regulation during acute illness [16,17]. Although these biomarkers have been associated with disease severity in some studies, their independent prognostic applicability remains uncertain. Most previous studies have assessed electrolyte disturbances or hormonal alterations separately. A comprehensive assessment of dysnatremia, thyroid-axis alterations, cortisol, vitamin D, insulin resistance, and GH/IGF-1 may provide a broader view of the endocrine–metabolic stress phenotype in hospitalized COVID-19 patients.

Therefore, the aims of this study were to describe the prevalence and clinical context of dysnatremia and endocrine alterations in hospitalized COVID-19 patients, to examine their associations with inflammatory, metabolic, and severity-related patterns, and to evaluate whether these parameters retain clinically meaningful prognostic value after adjustment for major clinical confounders.

Recent studies continue to characterize severe COVID-19 as a heterogeneous multisystem disease in which advanced age, pre-existing comorbidities, systemic inflammatory response, and acute organ dysfunction remain the principal determinants of adverse clinical outcomes [18,19]. Consequently, the incremental prognostic value of individual electrolyte or endocrine measurements should be assessed against these established clinical factors rather than inferred from unadjusted biomarker associations alone.

## 2. Materials and Methods

### 2.1. Study Design and Objectives

This was a prospective cohort study conducted at the Department of Infectious Diseases, University Hospital Centre Osijek, Croatia. Consecutive eligible patients were enrolled during hospitalization between January 2023 and December 2024. Clinical information and biological samples were collected prospectively at hospital admission according to a predefined study protocol, and clinical outcomes, including ICU admission and in-hospital mortality, were recorded during the index hospitalization. The study did not involve retrospective extraction of exposure or biomarker data from historical medical records.

Patients with known pre-existing electrolyte or endocrine alterations were excluded to focus on acute illness-related alterations. The primary aim was to determine the prevalence of dysnatremia and endocrine alterations at admission and to characterize their association with inflammatory, metabolic, and clinical features. A secondary aim was to assess whether sodium levels and hormonal parameters showed clinically meaningful prognostic value for disease severity, ICU admission, and in-hospital mortality after adjustment for relevant clinical confounders. The outcomes of interest were COVID-19 severity according to the WHO classification (mild, moderate, severe, and critical disease), ICU admission (yes/no), and in-hospital mortality (survivor/non-survivor).

The analyses were carried out hierarchically to reduce interpretation bias when testing several biomarkers. As an important and predetermined significant variable regarding the hypothesis of this study, serum sodium level was chosen as a main exposure. Secondary or exploratory variables included thyroid parameters as well as markers of glucose metabolism and a series of hormone assays.

The study protocol was approved by the Ethics Committee of the Clinical Hospital Centre Osijek and the Ethics Committee of the Faculty of Medicine Osijek. All procedures adhered to established ethical standards.

### 2.2. Participants

The analysis included hospitalized adults older than 18 years with confirmed SARS-CoV-2 infection and no known prior diagnosis of electrolyte or endocrine alterations. Patients were classified as SARS-CoV-2 positive if they had a positive rapid immunochromatographic test and/or PCR test. Participants were grouped according to admission sodium status and thyroid/hormonal findings into: (1) patients with dysnatremia and/or endocrine alterations and (2) patients with eunatremia and no endocrine alterations.

Patients were also stratified according to COVID-19 severity following the World Health Organization (WHO) classification into four categories: mild, moderate, severe, and critical COVID-19. Because several endocrine abnormalities occurred infrequently, the use of a composite endpoint allowed us to preserve statistical power and avoid multiple comparisons in a relatively small cohort. Patients with negative PCR results, known pre-existing electrolyte or endocrine alterations, or statin therapy were excluded.

Patients receiving statin therapy were excluded because of the potential effects of statins on metabolic and inflammatory parameters. However, because statin use is particularly common among older patients and patients with cardiovascular disease, diabetes, and other cardiometabolic comorbidities, this exclusion may have resulted in underrepresentation of higher-risk individuals and introduced selection bias. The findings should therefore not be assumed to be fully generalizable to an unselected population of hospitalized patients with COVID-19.

### 2.3. Methods and Laboratory Assays

Anamnestic data were collected for each participant, including age and sex, the day of illness at hospital admission, and information regarding SARS-CoV-2 testing (immunochromatographic or PCR test). Data on comorbidities were assessed using the Charlson Comorbidity Index, and clinical symptoms of COVID-19 were recorded, including general infectious, respiratory, neurological, and gastrointestinal manifestations.

A physical examination was conducted upon admission, including measurement of vital parameters such as body temperature, blood pressure, heart rate, oxygen saturation, and respiratory rate. At hospital admission, venous blood samples were collected from all patients using vacuum tubes. Blood for hematological analyses was collected into 3 mL BD Vacutainer® K2EDTA tubes, blood for coagulation analyses into 2.7 mL BD Vacutainer® 9NC trisodium citrate tubes, and blood for biochemical analyses into 4 mL BD Vacutainer® CAT clot activator tubes (Becton Dickinson and Company, Plymouth, UK). An additional serum sample was collected for the analysis of thyroid hormones (FT3, FT4), thyroid-stimulating hormone (TSH), cortisol, growth hormone, insulin-like growth factor 1 (IGF-1), and vitamin D. 

Complete and differential blood counts were analyzed using an automated Sysmex hematology analyzer (Sysmex Corporation, Kobe, Japan). Blood samples collected in sodium citrate tubes were centrifuged for 10 min at 2000× *g*, after which prothrombin time, D-dimer, fibrinogen, antithrombin III, and aPTT were measured in plasma using a BCS XP coagulation analyzer (Siemens Healthineers AG, Erlangen, Germany).

Serum samples collected in tubes without anticoagulant were prepared according to the manufacturer’s recommendations by centrifugation for 10 min at 2000× *g*. Serum urea, creatinine, AST, ALT, LDH, ALP, GGT, CK, and albumin were measured spectrophotometrically; CRP and ferritin were measured using immunoturbidimetric methods; and sodium, potassium, and chloride were measured by indirect potentiometry using a Beckman Coulter AU480 analyzer (Beckman Coulter, Inc., Brea, CA, USA), with the corresponding reagents used according to the manufacturer’s instructions.

Procalcitonin concentrations were measured using an electrochemiluminescence immunoassay (ECLIA) on a cobas e 611 immunoassay analyzer (Roche Diagnostics GmbH, Mannheim, Germany). High-sensitivity troponin I was determined using a homogeneous immunoassay based on luminescent oxygen channeling immunoassay (LOCI) technology on a Dimension EXL with LM Integrated Chemistry System (Siemens Healthcare Diagnostics Inc., Newark, DE, USA). Serum 25(OH)D concentrations were measured using an electrochemiluminescence method on a cobas 8000 modular analyzer (Roche Diagnostics, Basel, Switzerland). FT3, FT4, and TSH concentrations were determined using chemiluminescent immunoassays, whereas GH and IGF-1 concentrations were measured by radioimmunoassay using commercially available assay kits, including a human GH assay (DiaSorin S.p.A., Saluggia, Italy). All measurements were performed in the same clinical laboratory under standardized conditions, with established quality-control procedures and assay-specific reference ranges. Nevertheless, because these methods differ in their analytical principles, calibration, and sensitivity, some degree of measurement variability cannot be excluded. This should be considered when comparing results across different hormonal axes or with studies that used alternative analytical platforms.

Hormonal samples were obtained at hospital admission before corticosteroid administration and other therapeutic interventions. Because admissions occurred at different times of day, sampling was not uniformly restricted to the early morning. Accordingly, TSH and particularly cortisol values were interpreted as non-standardized admission measurements rather than standardized morning endocrine assessments.

Serum sodium and glucose were measured in the same admission blood sample before administration of intravenous fluids, corticosteroids, or other therapeutic interventions. Because hyperglycemia may lower measured serum sodium through osmotic water redistribution, sodium was corrected for concurrent glucose concentrations. The primary correction added 1.6 mmol/L to measured sodium for every 5.6 mmol/L increase in glucose above 5.6 mmol/L. A sensitivity analysis was performed using a correction factor of 2.4 mmol/L. Corrected sodium values were used for classification of hypo-, normo-, and hypernatremia and for all outcome analyses involving sodium status.

Serum sodium concentrations were classified after correction for hyperglycaemia. Hyponatremia was defined as a corrected serum sodium concentration < 135 mmol/L and further categorized as mild (130–134 mmol/L), moderate (125–129 mmol/L), and severe/profound (<125 mmol/L). Hypernatremia was defined as a corrected serum sodium concentration ≥ 146 mmol/L and categorized as mild (146–150 mmol/L), moderate (151–159 mmol/L), and severe (≥160 mmol/L). Dysnatremia was defined as the presence of either hyponatremia or hypernatremia based on glucose-corrected serum sodium concentrations.

During the study, clinical course of COVID-19 was monitored, including radiographic findings, development of complications, and the need for oxygen supplementation. Electrolyte levels and hormonal parameters were assessed at admission. Clinical outcomes were recorded, including discharge home, transfer to the intensive care unit, or death. All laboratory analyses were performed at the Department of Laboratory Diagnostics, University Hospital Centre Osijek. Microbiological analyses were conducted at the Department of Clinical Microbiology and Hospital Infections, and radiological examinations were performed at the Department of Diagnostic and Interventional Radiology within the same institution.

### 2.4. Sample Size and Statistical Analysis

A priori sample-size calculation indicated that at least 128 participants (64 per group) were required to detect the expected effect size with 80% power at a two-sided significance level of 0.05. Ultimately, 252 patients were enrolled, comprising 139 patients with dysnatremia and/or endocrine alterations and 113 controls, thus providing a substantially larger sample than initially required. The G*Power (version 3.1.2) calculation was based on the primary comparison between two independent groups.

Categorical variables are presented as absolute and relative frequencies. Differences between categorical variables were tested using the chi-square test. Normality of distribution for continuous variables was assessed using the Shapiro–Wilk test. Continuous variables are presented as median and interquartile range (IQR). Differences between two independent groups were assessed using the Mann–Whitney U test, with the difference, Hodges–Lehmann estimate, and corresponding 95% confidence intervals (95% CI) reported. Differences between three or more independent groups were assessed using the Kruskal–Wallis test with Conover post hoc analysis. Spearman’s rank correlation coefficient (ρ) was used to assess associations between variables. Multivariate logistic regression analysis was performed to identify factors associated with predefined clinical outcomes. Odds ratios (OR) with corresponding 95% confidence intervals are presented. 

Prior to multivariate logistic regression analysis, model assumptions were assessed by evaluating multicollinearity among predictors, linearity of continuous predictors with the logit of the outcome, and potential influential observations. Model calibration was additionally assessed using the Hosmer–Lemeshow goodness of fit test. ROC curve analysis was performed to assess the predictive performance of selected biomarkers and determine optimal cut-off values, area under the curve (AUC), sensitivity, and specificity for predicting in-hospital mortality. All *p* values were two-sided. The significance level was set at α = 0.05. Given the exploratory nature of secondary analyses, correction for multiple testing was not applied.

Serum sodium was treated as the principal exposure based on the prespecified study hypothesis. Analyses of additional hormonal and metabolic biomarkers, sex-stratified comparisons, correlation matrices, post hoc comparisons, and ROC-derived cut-off values were considered secondary and exploratory. No formal correction for multiple testing was applied. Consequently, nominal *p* values from these analyses should not be interpreted as confirmatory evidence, and statistically significant secondary findings may include false-positive results.

All sodium-related analyses were repeated using glucose-corrected sodium. Agreement between measured and corrected sodium categories was assessed by cross-tabulation, and the number and direction of reclassified cases were reported. Associations between corrected sodium categories and disease severity, ICU admission, and in-hospital mortality were assessed using appropriate categorical and regression analyses.

Differences in the distribution of hyponatremia and hypernatremia grades across COVID-19 severity and survival categories were evaluated using the χ^2^ test or Fisher–Freeman–Halton exact test when expected cell counts were small. Trends across ordered sodium-severity categories were evaluated using an exact trend test where applicable. Corrected sodium was additionally assessed as a continuous predictor. Multivariable logistic regression was performed only when the number of outcome events and observations per predictor was adequate. Results from sparse subgroups were considered descriptive and exploratory.

Analyses were performed using MedCalc^®^ Statistical Software version 23.5.5 (MedCalc Software Ltd., Ostend, Belgium), IBM SPSS Statistics version 23.0 (IBM Corp., Armonk, NY, USA), and Python version 3.12 (Python Software Foundation, Wilmington, DE, USA).

## 3. Results

### 3.1. Patient Characteristics

A total of 252 patients were included in the study, with a median age of 73 years (range 22–93) and a nearly equal sex distribution (50.4% female). The median duration of symptoms before hospital admission was 6 days (IQR 4–9), and the median length of hospital stay was 7 days (IQR 5–10). Overall, 40.9% of patients had received at least one COVID-19 vaccine dose (median 2 doses).

Comorbidities were highly prevalent (87.7%), most commonly arterial hypertension (81.0%), type 2 diabetes mellitus (36.2%), and cardiovascular disease (25.3%). Baseline demographic and clinical characteristics are summarized in Table 1.

### 3.2. Clinical Presentation, Laboratory Findings, and Disease Severity

**Figure A1:**
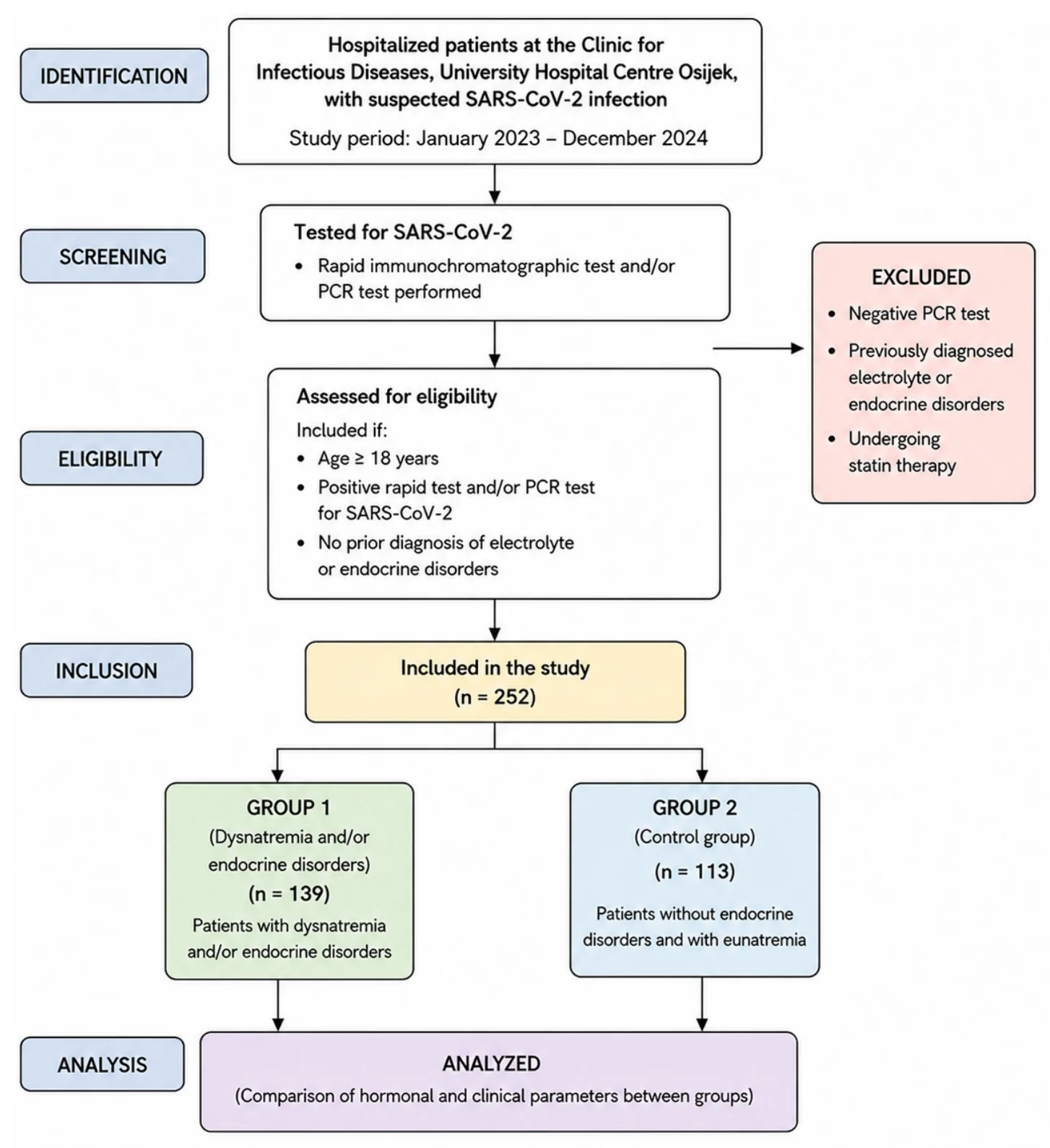
STROBE flow diagram of patient inclusion. Note: A total of 252 hospitalized adult patients with confirmed SARS-CoV-2 infection (January 2023–December 2024) were included. Patients were divided into Group 1 (dysnatremia and/or endocrine alterations, n = 139 [55.2%]) and Group 2 (control group, n = 113 [44.8%]). Patients with negative PCR results, prior electrolyte or endocrine alterations, or on statin therapy were excluded. Abbreviations: PCR, polymerase chain reaction; SARS-CoV-2, severe acute respiratory syndrome coronavirus 2.

The most common symptoms at admission were cough (74.2%), fever (70.2%), and dyspnea (47.2%). Pneumonia was present in 89.7% of patients, predominantly bilateral pneumonia (82.3%). Pleuropneumonia was observed in 27.4% of patients, while pulmonary embolism was documented in 2.4%. The cohort represented a clinically severe hospitalized population. Oxygen supplementation was required in 86.9% of patients, most commonly via face mask (42.1%), followed by nasal catheter (19.8%), high-flow nasal cannula (16.7%), and non-invasive ventilation (2.8%). Invasive mechanical ventilation was required in 6.3% of patients, and multi-organ failure occurred in 7.5%. According to COVID-19 severity classification, severe disease was the most common presentation, followed by critical and moderate disease, while mild disease was rare. At admission, patients showed marked inflammatory and laboratory abnormalities, including elevated CRP, IL-6, and D-dimer concentrations, as well as lymphopenia. The median CRP concentration was 77.1 mg/L, median IL-6 was 58.2 pg/mL, and median D-dimer concentration was 1420.5 ng/mL. Median lymphocyte percentage was 13%, indicating a frequent inflammatory-cell pattern consistent with acute systemic illness. Patient inclusion, exclusion, and grouping are shown in Figure A1 (Appendix A).

### 3.3. Prevalence of Dysnatremia and Endocrine Alterations

Dysnatremia and/or endocrine alterations were frequent in this hospitalized COVID-19 cohort. Overall, 139 patients (55.2%) had dysnatremia and/or endocrine alterations, whereas 113 patients (44.8%) had normal sodium levels and no endocrine alterations. Hyponatremia was observed in 42 patients (16.7%), while hypernatremia was present in 23 patients (9.1%).

Thyroid-function alterations consistent with non-thyroidal illness syndrome were observed in 103 patients (40.9%), most commonly as decreased or suppressed TSH concentrations. The prevalence of the main sodium and endocrine abnormalities is summarized in Table 2.

**Figure A2:**
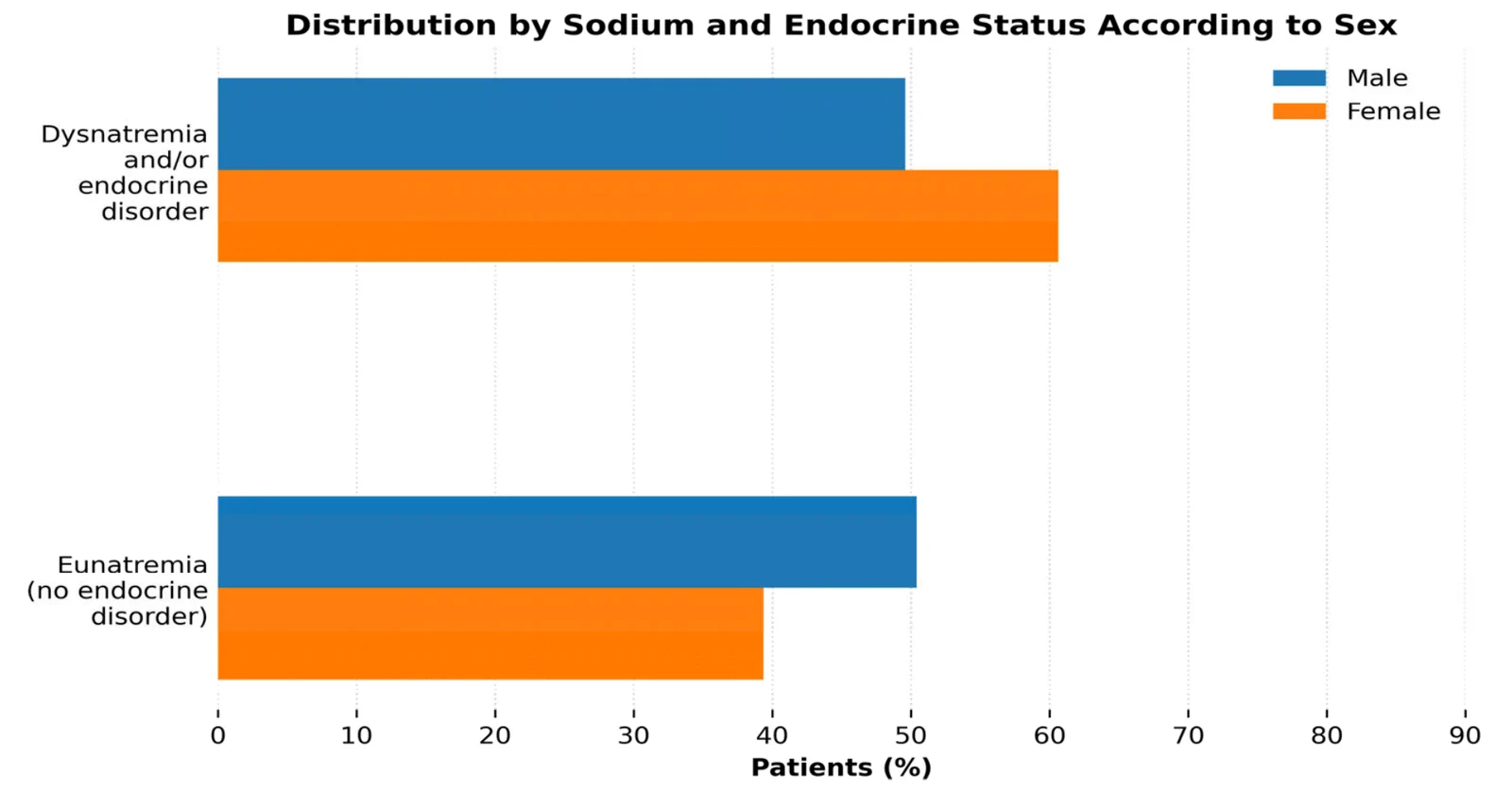
Distribution of patients according to sodium and endocrine status by sex. Note: The distribution of dysnatremia and/or endocrine alterations did not differ significantly between male and female patients (chi-square test, *p* = 0.102).

The distribution of sodium/endocrine status did not differ significantly between female and male patients, as shown in Figure A2 (Appendix A).

### 3.4. Dysnatremia-Associated Clinical and Laboratory Patterns

After correction of serum sodium for hyperglycemia, no significant differences in clinical or laboratory characteristics were observed between patients with dysnatremia and those with normal sodium concentrations.

However, sodium concentrations did not differ significantly according to disease-severity category, ICU admission status, or survival outcome. Median sodium concentrations were similar across disease-severity groups, ICU and non-ICU patients, and survivors and non-survivors. These findings suggest that dysnatremia was associated with metabolic and inflammatory stress patterns but did not function as an independent marker of clinical outcome in this cohort.

Hormonal and biochemical parameters according to COVID-19 severity are shown in Table 3. Because only one patient had mild disease, comparisons across severity categories should be interpreted cautiously and mainly reflect differences between moderate, severe, and critical disease.

Measured and glucose-corrected serum sodium concentrations for the overall cohort are presented in Table 4. After correction for concurrent glycemia, 42 patients (16.7%) had hyponatremia, 187 (74.2%) had normonatremia, and 23 (9.1%) had hypernatremia. Correction changed the sodium category in 15 patients (6.0%) (Table 5). Because of the small numbers of patients with moderate and severe hypernatremia, these findings should be interpreted cautiously.

Sodium concentrations did not differ significantly according to COVID-19 disease severity (Table 6).

Among patients with hyponatremia, 34 had mild, 6 had moderate, and 2 had severe hyponatremia, whereas among patients with hypernatremia, 14 had mild, 6 had moderate, and 3 had severe hypernatremia (Table 7). The distribution of hyponatremia grades did not differ according to COVID-19 severity (*p* = 0.78; Table 8) or survival status (*p* > 0.99; Table 9). Likewise, the distribution of hypernatremia grades did not differ according to COVID-19 severity (*p* = 0.34; Table 10) or mortality (*p* = 0.48; Table 11). Because the number of hypernatremic patients was small, analyses by hypernatremia grade were primarily descriptive, and exact statistical methods were used where appropriate. We avoided overfitted regression models in categories containing very few events. The mild COVID-19 category included only one patient and was therefore excluded from statistical analyses because meaningful comparisons could not be performed.

Sodium concentrations did not differ significantly between patients who required ICU admission and those who did not, either in the overall cohort or after stratification by sex (Table 12).

Sodium concentrations were significantly higher in deceased than in surviving patients in the overall cohort (Mann–Whitney U test, *p* = 0.04), although the estimated median difference was small (1.37 mmol/L). However, no significant differences in sodium concentrations were observed between deceased and surviving patients when analyses were stratified by sex (Table 13).

### 3.5. Thyroid Function and Non-Thyroidal Illness Syndrome

**Figure 1:**
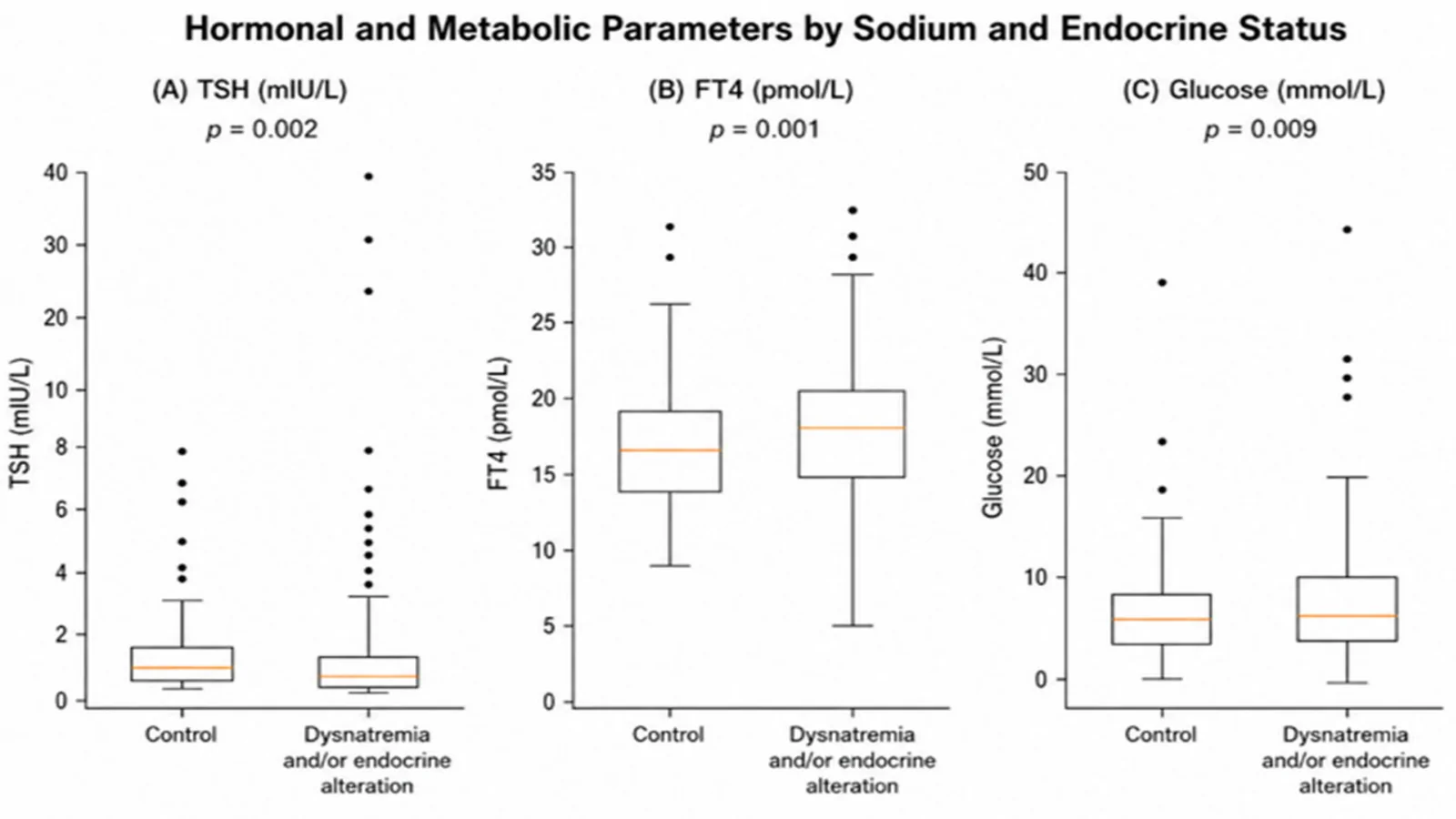
Hormonal and metabolic parameters according to sodium and endocrine status. Box plots compare serum TSH (**A**), FT4 (**B**), and glucose (**C**) concentrations between the control group and patients with dysnatremia and/or endocrine alterations. Compared with controls, the dysnatremia/endocrine alteration group had lower TSH (*p* = 0.002), higher FT4 (*p* = 0.001), and higher glucose concentrations (*p* = 0.009). Boxes represent the interquartile range (IQR), the central line indicates the median, whiskers extend to 1.5 × IQR, and dots represent outliers. Abbreviations: TSH, thyroid-stimulating hormone; FT4, free thyroxine. Units: mIU/L, milli-international units per liter; pmol/L, picomoles per liter; mmol/L, millimoles per liter.

Thyroid-function alterations consistent with non-thyroidal illness syndrome were found in 40.9% of patients. Euthyroidism was observed in 59.1%. The dominant pattern was decreased or suppressed TSH concentration, observed in 39.8% of patients. Given the acute COVID-19 setting and the absence of follow-up thyroid testing after recovery, these findings should not be interpreted as evidence of subclinical hyperthyroidism, but rather as acute illness-related thyroid-axis alterations. Patients with dysnatremia and/or endocrine alterations had significantly lower TSH levels than patients with normal sodium and endocrine status (*p* = 0.006). They also had significantly higher FT4 levels (*p* = 0.002). Glucose levels showed a borderline difference between the groups (*p* = 0.05). In female patients, dysnatremia and/or endocrine alterations were associated with significantly lower TSH concentrations (*p* = 0.01) (*p* = 0.02) and higher FT4 concentrations (*p* = 0.004). These differences are illustrated in Figure 1.

Most thyroid parameters did not differ significantly across disease-severity categories. Median TSH concentrations ranged from 0.8 to 0.9 mIU/L across severity groups (*p* = 0.84), median FT4 concentrations from 17.0 to 17.6 pmol/L (*p* = 0.34), and median FT3 concentrations from 3.0 to 3.1 pmol/L (*p* = 0.24). Similarly, no significant differences in TSH or FT3 were observed according to ICU admission status. In mortality analyses, deceased patients had significantly lower FT3 levels than survivors (2.8 [2.1–3.5] vs. 3.2 [2.7–4.0] pmol/L; difference −0.5, 95% CI −0.7 to −0.2; *p* < 0.001). Among female patients, mortality was additionally associated with lower TSH concentrations (0.7 [0.3–1.6] vs. 1.2 [0.5–2.2] mIU/L; difference −0.4, 95% CI −0.8 to −0.04; *p* = 0.03) and lower FT3 concentrations (2.7 [2.1–3.1] vs. 3.1 [2.6–3.9] pmol/L; difference −0.5, 95% CI −0.8 to −0.2; *p* = 0.002).

### 3.6. Metabolic Parameters

Among disease-severity categories, insulin was the only metabolic parameter that differed significantly. Insulin concentrations were higher in patients with severe disease than in those with moderate disease (90.6 [45.4–155.5] vs. 51.1 [25.6–108] pmol/L; *p* = 0.02). Glucose concentrations and HOMA-IR values did not differ significantly according to disease severity. Patients admitted to the ICU had significantly higher glucose levels than non-ICU patients (7.0 [5.3–10.7] vs. 6.0 [4.7–8.6] mmol/L; difference 0.97, 95% CI 0.04–1.9; *p* = 0.04). HOMA-IR values were also higher in ICU patients (29.4 [11.7–75.1] vs. 17.4 [5.9–39.0]; difference 10.1, 95% CI 0.8–21.8; *p* = 0.02). In sex-stratified analyses, these associations were mainly observed in female patients, in whom ICU admission was associated with higher glucose levels (7.6 [5.6–12.4] vs. 6.2 [4.5–8.7] mmol/L; *p* = 0.03). No consistent significant metabolic differences were observed in male patients. Hormonal and metabolic parameters according to ICU admission are shown in Table 14. The extended version of the ICU admission analysis, including GH, IGF-1, and insulin, is provided in Table A1 (Appendix A).

### 3.7. Other Endocrine Parameters

Most hormonal parameters, including cortisol, GH, IGF-1, and vitamin D3, did not differ significantly across disease-severity categories or according to ICU admission status. However, several endocrine markers were associated with mortality in univariate analyses. Deceased patients had significantly higher cortisol concentrations than survivors (684.5 [495–1015.8] vs. 555 [376–895] nmol/L; difference 136, 95% CI 24–247; *p* = 0.02). They also had significantly lower vitamin D3 levels (21.5 [12.9–34.2] vs. 29.7 [17.5–42.9] nmol/L; difference −6.7, 95% CI −11.7 to −2.1; *p* = 0.003).

Among female patients, vitamin D3 concentrations were markedly lower in deceased patients than in survivors (15.6 [11.8–25.2] vs. 28.7 [17.1–40.0] nmol/L; difference −8.1, 95% CI −14.6 to −2.2; *p* = 0.008).

Hormonal and metabolic parameters according to survival status are shown in Table 15. The extended survival analysis is provided in Table A2 (Appendix A).

### 3.8. Sex-Specific Correlation Analysis

**Figure 2:**
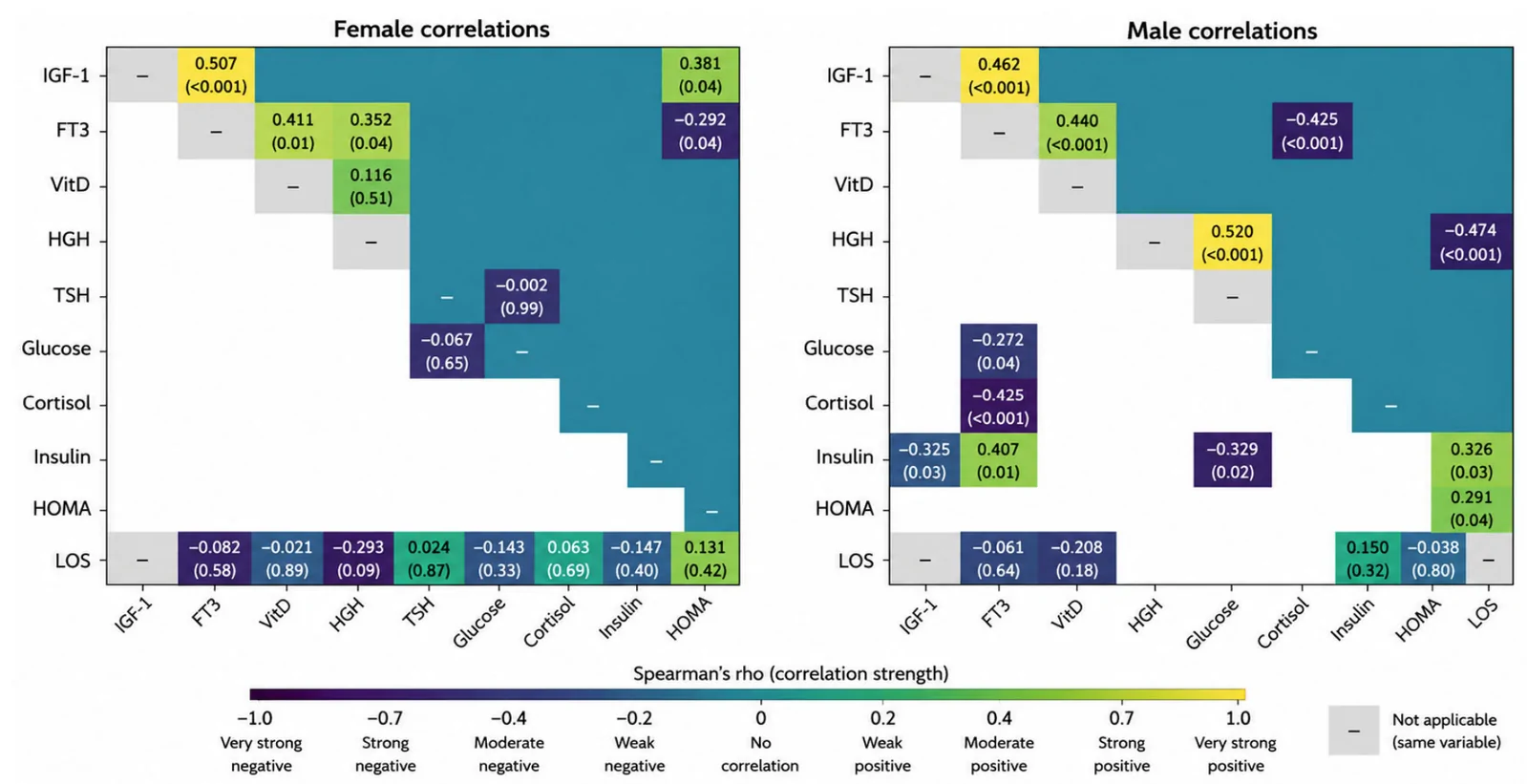
Sex-specific correlation heatmaps of endocrine, metabolic, and clinical parameters. Heatmaps show Spearman correlation coefficients between selected variables in female and male patients. Color intensity indicates the strength and direction of correlations. Only statistically significant correlations are displayed. Abbreviations: IGF-1, insulin-like growth factor 1; FT3, free triiodothyronine; VitD, vitamin D; HGH, human growth hormone; TSH, thyroid-stimulating hormone; HOMA, homeostatic model assessment; LOS, length of stay.

Sex-specific correlation analyses suggested distinct endocrine-metabolic clustering in female and male patients. In female patients, the strongest correlations were observed between IGF-1 and FT3 (rho = 0.507; *p* < 0.001), vitamin D3 and FT3 (rho = 0.411; *p* = 0.01), and GH and FT3 (rho = 0.352; *p* = 0.04). In male patients, the strongest correlations included IGF-1 and FT3 (rho = 0.462; *p* < 0.001), cortisol and FT3 (rho = −0.425; *p* < 0.001), insulin and cortisol (rho = −0.435; *p* < 0.001), insulin and sodium (rho = −0.326; *p* = 0.03), and HOMA-IR and GH (rho = −0.457; *p* < 0.001). These sex-specific endocrine, metabolic, and clinical correlations are shown in Figure 2.

### 3.9. ROC Analysis

ROC analyses demonstrated statistically significant but modest discriminative performance for selected hormonal and metabolic parameters. In female patients, FT4 showed modest discrimination for severe/critical disease, with an AUC of 0.639 (95% CI 0.549–0.722; sensitivity 82%; specificity 42%; Youden index 0.237; cut-off <= 16.8; *p* = 0.02). Insulin also showed modest discrimination for severe/critical disease in female patients, with an AUC of 0.644 (95% CI 0.534–0.743; sensitivity 71%; specificity 58%; Youden index 0.290; cut-off > 71.4; *p* = 0.04).

For mortality, TSH and FT3 showed statistically significant but weak discriminative ability. TSH had an AUC of 0.623 (95% CI 0.533–0.707; *p* = 0.02), while FT3 had an AUC of 0.671 (95% CI 0.582–0.752; *p* < 0.001). Vitamin D3 demonstrated modest discriminative ability for mortality in female patients, with an AUC of 0.655 (95% CI 0.562–0.740; sensitivity 56%; specificity 78%; Youden index 0.333; cut-off <= 25.3; *p* = 0.006).

**Figure 3:**
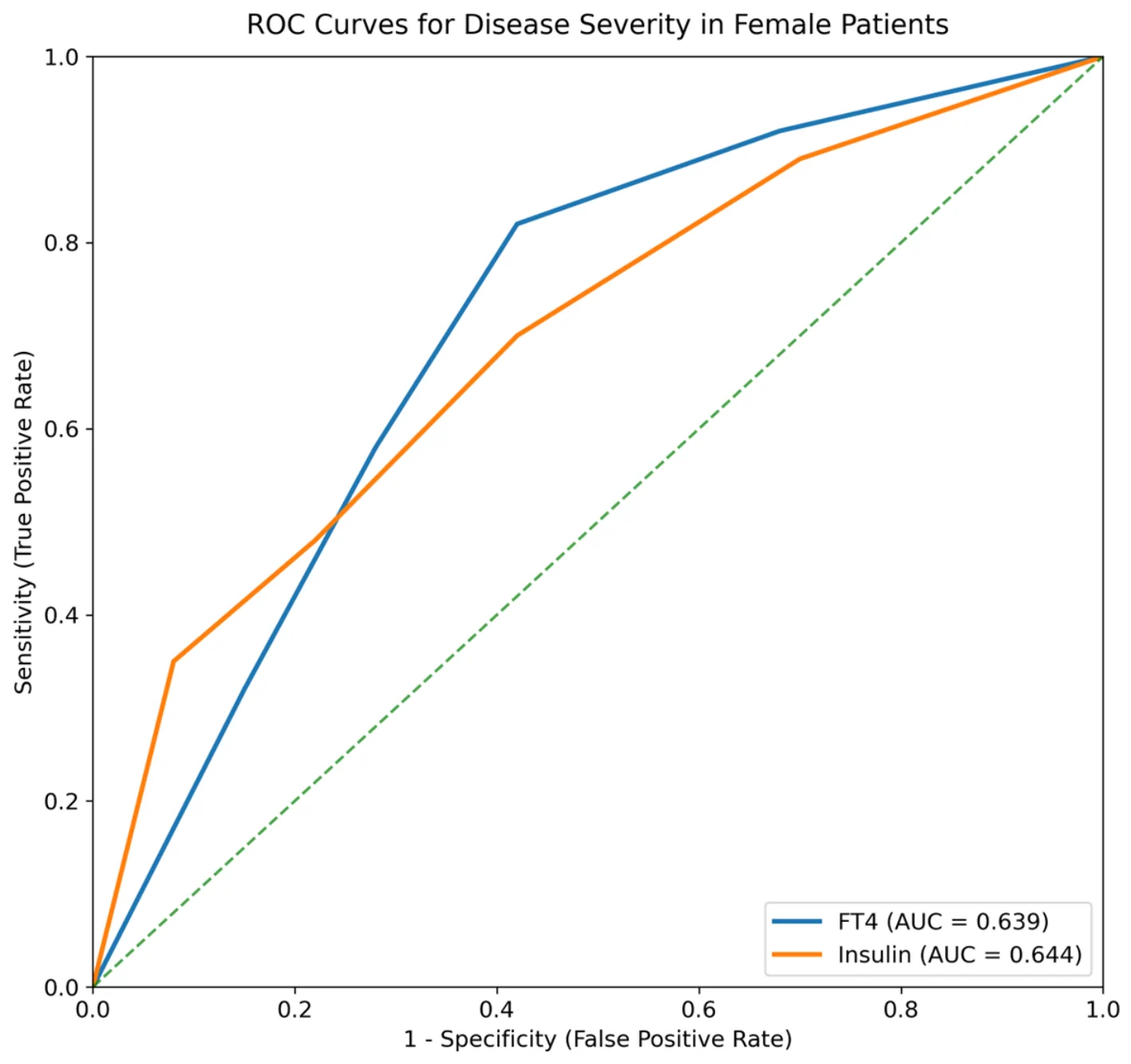
ROC curves for disease severity. Note: ROC analyses showed that selected hormonal and metabolic parameters had statistically significant but weak discriminative ability for disease severity and mortality. In female patients, FT4 and insulin showed modest discrimination for severe/critical disease, while TSH, FT3, and vitamin D3 showed modest discrimination for mortality. Abbreviations: ROC, receiver operating characteristic; AUC, area under the curve; FT4, free thyroxine.

Although several ROC analyses reached nominal statistical significance, all AUC values were below 0.70. This represents poor or, at best, modest discrimination and indicates that none of the evaluated biomarkers had sufficient standalone performance for individual prediction of disease severity or mortality. The reported cut-off values were data-derived exploratory thresholds and should not be interpreted as clinically validated decision limits. ROC curves are shown in Figure 3.

### 3.10. Multivariable Analyses

A total of 51 patients (20.2%) required ICU admission. Overall, 184 patients (73.0%) survived, while 68 patients (27.0%) died during hospitalization. In multivariable logistic regression models adjusted for age, sex, hypertension, type 2 diabetes, admission glucose, and admission CRP, dysnatremia and/or endocrine alterations were not independently associated with severe/critical disease, ICU admission, or in-hospital mortality. Similarly, most individual clinical and biochemical predictors did not retain independent prognostic significance after adjustment.

In the ICU admission model, admission glucose showed a statistically significant association with ICU admission, with each 1 mmol/L increase in glucose associated with 7% higher odds of ICU treatment (OR 1.07; 95% CI 1.01–1.13; *p* = 0.02). No independent association was observed between dysnatremia and/or endocrine alterations and ICU admission. In the mortality model, age showed borderline statistical significance, while dysnatremia and/or endocrine alterations were not independently associated with death.

**Figure 4:**
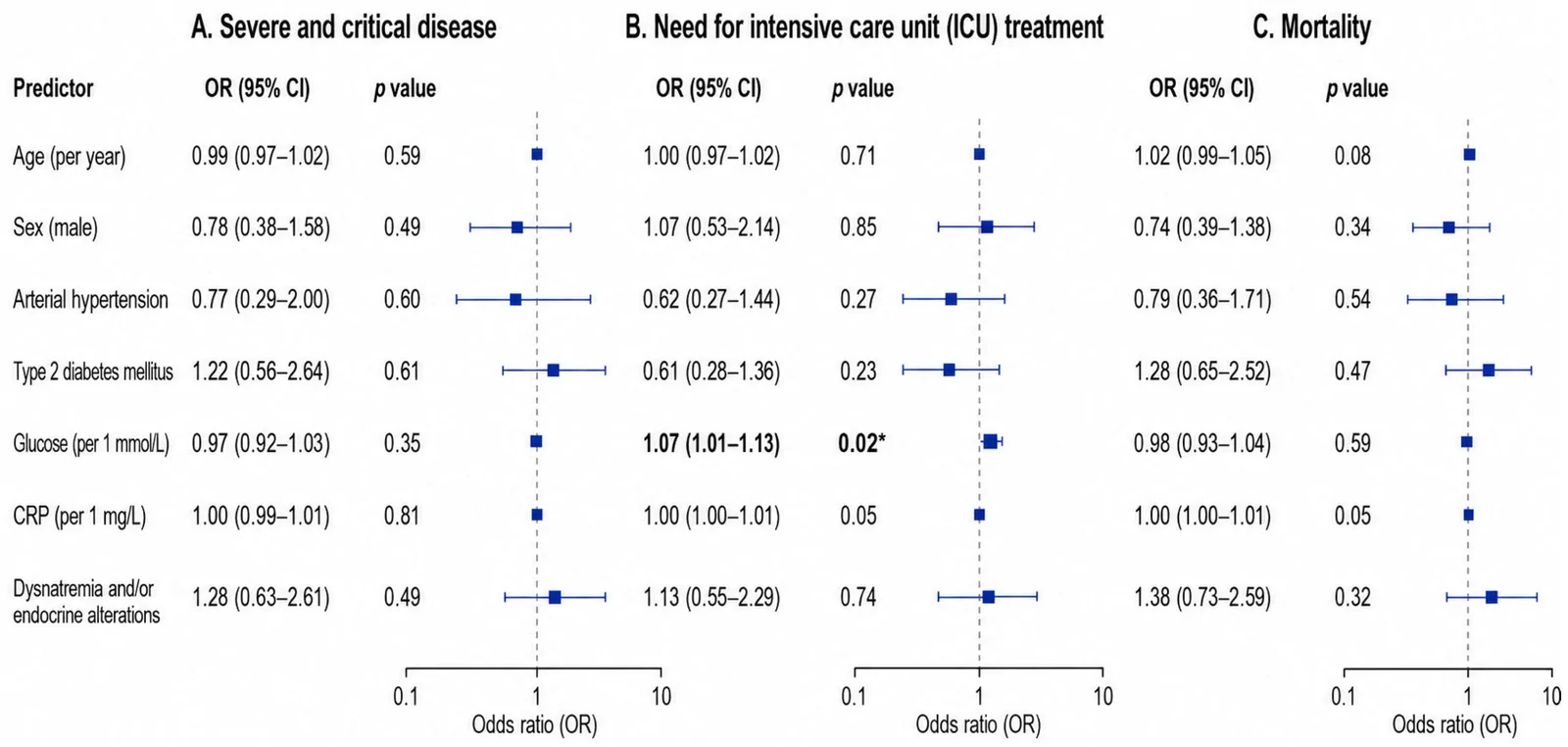
Forest plots of multivariate logistic regression models for severe and critical disease, ICU admission, and mortality. Squares represent odds ratios, and horizontal lines represent 95% confidence intervals. The vertical dashed line indicates an odds ratio of 1. Higher glucose levels were independently associated with ICU admission, whereas dysnatremia and/or endocrine alterations were not independently associated with any of the examined outcomes. * *p* < 0.05. Abbreviations: OR, odds ratio; CI, confidence interval; CRP, C-reactive protein.

The multivariable logistic regression models are presented in Figure 4. The detailed multivariable model for severe and critical diseases is presented in Table A3 (Appendix A).

## 4. Discussion

### 4.1. Main Findings

The principal finding of this study is not that dysnatremia or endocrine alterations independently predict adverse outcomes, but that they frequently accompany the systemic response to acute COVID-19. Several parameters were associated with outcomes in unadjusted or subgroup analyses, but most associations did not persist after adjustment for major clinical and inflammatory confounders. This attenuation suggests that the biomarkers largely capture overlapping information related to age, comorbidity burden, inflammation, metabolic stress, and acute illness severity rather than providing independent prognostic information. Accordingly, the absence of independent associations in the adjusted models should be given greater interpretative weight than isolated nominally significant findings from unadjusted, subgroup, or ROC analyses.

### 4.2. Dysnatremia

Dysnatremia was highly prevalent in our cohort but was not independently associated with mortality or other adverse clinical outcomes after adjustment for potential confounders. These changes are almost certainly overwhelmingly the host’s response to critical acute illness under the combined stress of inflammation, neurohormonal activation, metabolic stress, underlying comorbidities, and treatment. Therefore, dysnatremia likely reflects the complex pathophysiological processes associated with severe illness and may serve as an integrated marker of disease severity rather than an independent determinant of clinical outcomes. Various studies have demonstrated that a considerable number of hospitalized patients with hyponatremia, which correlates with escalated disease severity, a higher demand for intensive care, and increased mortality rates [20,21,22]. Proposed mechanisms of hyponatremia in COVID-19 include SIADH, adrenal insufficiency, and hypovolemic sodium loss related to gastrointestinal fluid depletion [4,23]. In our cohort, dysnatremia was associated with elevated glucose concentrations. These findings are concordant with the interpretation that sodium imbalance accompanies inflammatory activation, metabolic stress, and acute illness physiology rather than acting as an isolated driver of unfavorable outcomes.

Reanalysis using glucose-corrected sodium was necessary because patients categorized as dysnatremic had higher glucose concentrations than those with normal sodium. Hyperglycemia may lower measure sodium through osmotic water redistribution and can therefore produce apparent or exaggerated hyponatremia. After glucose correction, the prevalence of hyponatremia decreased from 21.0% (53/252) to 16.7% (42/252), whereas the prevalence of hypernatremia increased from 6.3% (16/252) to 9.1% (23/252), resulting in reclassification of sodium status in 15 patients (6.0%). Despite these changes, sodium was not independently associated with severe or critical disease, ICU admission, or mortality in multivariable analyses.

The grading analysis further showed that most cases of hyponatremia were mild (34/42, 81.0%), followed by moderate (6/42, 14.3%), whereas only two patients (4.8%) had severe hyponatremia. Hypernatremia was less frequent and occurred predominantly in its mild form (14/23, 60.9%), with only three patients (13.0%) meeting the criteria for severe hypernatremia. No significant associations were observed between the severity of sodium disturbances and COVID-19 severity, ICU admission, or mortality. However, the limited number of patients in the more extreme sodium categories reduced statistical precision and precluded firm conclusions regarding dose–response relationships.

These findings indicate that the clinical interpretation of sodium abnormalities in acute COVID-19 should consider both concurrent glucose concentrations and the severity of the sodium disturbance. Recent data from Piza et al. demonstrated that hyponatremia is associated with adverse outcomes in hospitalized patients with COVID-19, supporting the concept that sodium disturbances primarily reflect the severity of the underlying systemic illness and the integrated stress response rather than representing isolated electrolyte abnormalities or independent prognostic factors [22]. In our cohort, failure to correct sodium serum for hyperglycemia would have resulted in misclassification of sodium status, particularly by overestimating hyponatremia. Glucose correction reclassified sodium status in 6.0% of patients, primarily by reducing the prevalence of hyponatremia, yet sodium was not independently associated with disease severity, ICU admission, or mortality after multivariable adjustment.

### 4.3. Endocrine Alterations

Endocrine abnormalities were also common and were most notably characterized by changes in thyroid function consistent with non-thyroidal illness syndrome (NTIS). Importantly, because patients with pre-existing endocrine disorders were excluded, these alterations are unlikely to represent chronic endocrine disease and instead most likely reflect adaptive changes within endocrine axes during acute illness. In our study most hormonal parameters—including vitamin D, IGF-1, and cortisol—did not differ significantly across disease-severity categories. Insulin levels differed across disease-severity categories, and higher glucose and HOMA-IR values were observed in patients requiring ICU admission. These findings may indicate stress-related alterations in glucose metabolism during acute COVID-19 illness rather than chronic metabolic insulin resistance. Nevertheless, neither parameter showed independent prognostic significance in multivariable analyses. Vitamin D and IGF-1 have been proposed as potential biomarkers in COVID-19; however, their clinical significance remains inconsistent across studies [24,25]. In the present cohort, neither marker demonstrated meaningful independent prognostic value, supporting the interpretation that these parameters primarily reflect general health status and acute systemic stress rather than disease-specific mechanisms. Cortisol, a main stress hormone from the HPA system, is elevated during acute illness and has been linked to severity and mortality of COVID-19 [13,26]. Higher levels of cortisol have also been seen with inflammatory burden, mechanical ventilatory support and worse clinical outcomes [27]. In our study, no significant differences in cortisol were noted between disease severity groups, possibly because timing of measurement, disease stage, the specific patients’ stress response or the use of steroids influenced individual values. Individual admission values alone also may not adequately capture the variation in endocrine and metabolic change that takes place during acute illness. In our cohort, deceased patients had higher admission cortisol concentrations and lower FT3 concentrations than survivors in the unadjusted analysis, findings consistent with an acute systemic stress response and a non-thyroidal illness pattern. However, because blood sampling was not standardized with respect to time of day and post-recovery thyroid function was not assessed, these observations should be considered exploratory. Furthermore, the observed associations did not retain independent significance after adjustment and therefore cannot be interpreted as evidence of prognostic or diagnostic utility. These results could suggest that cortisol and associated hormones may indicate general stress and response to treatment rather than being predictive of a specific disease process [28].

Although each hormonal parameter was measured using a consistent analyte-specific method, the use of different analytical platforms across hormonal axes may have contributed to heterogeneity in analytical precision, calibration, sensitivity, and reference ranges. Such variability could have attenuated true associations or contributed to weak nominal associations, particularly for GH and IGF-1, which may also show substantial biological variability during acute illness. These findings should therefore not be directly extrapolated to cohorts assessed using different assay platforms.

### 4.4. Sex Differences and ROC Analysis

Several biomarkers, including FT3, TSH, and vitamin D3, demonstrated statistically significant associations with mortality in female patients. These findings are consistent with previous reports suggesting sex-specific differences in endocrine and metabolic adaptation to severe illness [26,29,30]. These results align with existing literature indicating that thyroid hormones, especially FT3, are indicative of the severity of systemic illness and the burden of inflammation, rather than acting as robust standalone prognostic indicators. Decreased levels of FT3 have been correlated with an elevated risk of mortality across diverse populations; however, their ability to discriminate remains constrained, as they primarily reflect adaptive metabolic responses to illness instead of serving as causal predictors of outcomes [31,32]. Likewise, while hypovitaminosis D is associated with negative outcomes and death in many diverse clinical situations, it is not a distinct prognostic marker. Previous studies in other acute conditions, including heart failure, have demonstrated poor discrimination for mortality prediction despite statistically significant associations [33]. Overall, our findings suggest that endocrine and electrolyte disturbances in COVID-19 reflect integrated stress physiology rather than isolated organ dysfunction, which limits their isolated prognostic value. Hence, their maximal value could reside in the phenotype: they help characterize the biological state of hospitalized patients and should be interpreted alongside the broader clinical and inflammatory context.

Although some ROC analyses were statistically significant, all AUC values remained below 0.70, suggesting only limited discriminative ability. Consequently, the investigated biomarkers are unlikely to provide sufficient predictive accuracy when used alone for individual risk stratification. The proposed cut-off values should therefore be regarded as exploratory and warrant external validation before any clinical application.

### 4.5. Strengths and Limitations

A major strength of this study is its prospective design with systematic assessment of hospitalized COVID-19 patients at admission. The study combined electrolyte, endocrine, metabolic, inflammatory, and clinical data and linked them to relevant outcomes, including disease severity, ICU admission, and in-hospital mortality. Unlike several previous COVID-19 studies that classified dysnatremia according to measured serum sodium concentrations without accounting for concomitant hyperglycemia, our study systematically used glucose-corrected sodium values, thereby reducing the risk of misclassifying dilutional hyponatremia [34,35,36].

Another strength is the broad endocrine–metabolic profiling, which allowed evaluation of dysnatremia and hormonal alterations as part of an integrated stress phenotype rather than as isolated biomarkers. The use of sex-stratified analyses, ROC curves, and multivariable models adjusted for major clinical confounders further strengthened the interpretation of the findings. This study also has limitations. Biomarkers were assessed only at hospital admission, preventing evaluation of their dynamic changes during hospitalization or recovery. The single-center design may limit generalizability.

In addition, exclusion of patients receiving statin therapy may have reduced cohort representativeness by underrepresenting individuals with a high cardiometabolic burden. Additionally, the composite outcome combining dysnatremia and endocrine alterations included heterogeneous abnormalities with potentially different clinical implications; however, separate analyses were limited by the relatively small sample size and low frequency of individual disorders.

Hormonal blood sampling was performed at hospital admission and was not uniformly standardized to an early-morning interval. Circadian variation may therefore have influenced cortisol and TSH concentrations and increased measurement variability. This limitation is particularly relevant for cortisol, for which a single non-timed value cannot be interpreted as a standardized assessment of hypothalamic–pituitary–adrenal function. It may also have contributed to lower TSH values, although it is less likely to fully explain the observed FT3 pattern. Consequently, these hormonal findings should be considered exploratory and contextual rather than diagnostic or predictive.

Multiple biomarkers, outcomes, subgroup comparisons, sex-stratified analyses, correlations, and ROC curves were evaluated without formal adjustment for multiple testing. This increases the probability of type I errors, particularly for findings with *p* values close to 0.05. Therefore, secondary statistically significant findings should be regarded as exploration and hypothesis-generating rather than confirmatory. The results require replication in an independent cohort with prespecified hypotheses and appropriate control of the false discovery rate or family-wise error rate.

The observed dysnatremia and endocrine abnormalities appear to have primarily contextual rather than predictive clinical value. They reflect the endocrine–metabolic response to acute systemic illness and may contribute to the characterization of the patient’s physiological stress state. However, they do not provide sufficiently accurate or independent information for individual risk stratification and should always be interpreted in conjunction with age, comorbidity burden, respiratory status, inflammatory markers, renal function, medications, and the overall clinical course.

Despite these limitations, the prospective design, broad biomarker assessment, outcome-based analyses, and adjusted statistical approach strengthen the main conclusion that electrolyte and endocrine abnormalities in hospitalized COVID-19 patients are common and clinically informative, but should primarily be interpreted as markers of systemic stress physiology rather than as standalone prognostic biomarkers.

## 5. Conclusions

Dysnatremia and endocrine alterations were common among hospitalized patients with COVID-19 and were associated with metabolic and inflammatory disturbances, reflecting the complex systemic response to acute infection. However, the discriminatory performance of the investigated biomarkers was limited, with all AUC values below 0.70, and neither dysnatremia nor endocrine alterations independently predicted disease severity, ICU admission, or mortality after multivariable adjustment. These findings suggest that electrolyte and endocrine abnormalities should be interpreted primarily as markers of systemic illness and the integrated stress response rather than as independent prognostic biomarkers. Although their identification may contribute to clinical phenotyping and highlight abnormalities requiring appropriate evaluation and management, they should not be used in isolation for individual risk stratification. Furthermore, because hormonal measurements were obtained at a single time point and the timing of sampling was not standardized, our findings do not support the use of admission TSH or cortisol concentrations as standalone diagnostic or prognostic endocrine markers in hospitalized patients with COVID-19.

Future longitudinal, treatment-adjusted, and sex-aware studies are needed to determine whether trajectories of endocrine and electrolyte changes, rather than single admission measurements, may improve risk stratification in hospitalized patients with acute systemic illness.

## Figures and Tables

**Table 1:** Baseline characteristics of the study population.

Characteristic	Total (n = 252)
Sex [n (%)]	
Female	127 (50.4)
Male	125 (49.6)
Age [Median (IQR), years]	73 (63–80.8)
Duration of illness at admission [Median (IQR), days]	6 (4–9)
Length of hospital stay [Median (IQR), days]	7 (5–10)
Vaccinated [n (%)]	103 (40.9)
Number of vaccine doses [Median (IQR)]	2 (2–2)
Comorbidities [n (%)]	221 (87.7)
Arterial hypertension	179 (81.0)
Type 2 diabetes mellitus	80 (36.2)
Cardiovascular disease	56 (25.3)
Previous cerebrovascular stroke	34 (15.4)
Atrial fibrillation	33 (14.9)

Data are presented as median (interquartile range) or number (percentage). Abbreviations: IQR, interquartile range.

**Table 2:** Prevalence of dysnatremia and endocrine alterations.

Alteration	n (%)
Dysnatremia and/or endocrine alteration	139 (55.2)
Normal sodium and no endocrine alteration	113 (44.8)
Hyponatremia	42 (16.7)
Hypernatremia	23 (9.1)
Thyroid-function alteration/NTIS pattern	103 (40.9)
Decreased or suppressed TSH concentration	100 (39.8)

Abbreviations: NTIS, non-thyroidal illness syndrome; TSH, thyroid-stimulating hormone.

**Table 3:** Hormonal and biochemical parameters according to disease severity.

Parameter	Median (IQR) According to Disease Severity				*p* *
	Mild (n = 1)	Moderate (n = 45)	Severe (n = 159)	Critical (n = 48)	
	(1)	(2)	(3)	(4)	
Sodium (mmol/L)	138.0	140 (137–143)	139 (136–141)	138 (136–141)	0.32
TSH [mIU/L]	1.5	0.9 (0.5–1.3)	0.9 (0.5–1.7)	0.8 (0.4–1.9)	0.84
FT4 [pmol/L]	22.3	17.4 (15.4–20.3)	17.6 (15–20.3)	17 (14.1–20.1)	0.34
FT3 [pmol/L]	5.8	3.1 (2.4–3.8)	3.1 (2.6–3.8)	3 (2.5–3.6)	0.24
Cortisol [nmol/L]	296.0	578.5 (314.5–856.5)	603 (412.8–921.8)	606.5 (403.8–847.8)	0.67
GH [ng/mL]	1.2	0.6 (0.3–1.5)	0.5 (0.2–1.2)	0.5 (0.2–1)	0.56
IGF-1 [nmol/L]	19.0	7.7 (4.2–11.5)	8 (4.7–11.8)	8 (5.4–12.2)	0.48
Insulin [pmol/L]	109.0	51.1 (25.6–108)	90.6 (45.4–155.5)	66.3 (31–128)	0.02 †
Vitamin D3 [nmol/L]	30.0	27.7 (12.5–41.4)	26.8 (16.9–39.3)	29.1 (15.7–44.2)	0.77
Glucose [mmol/L]	4.4	5.9 (4.7–11.3)	6.4 (5–8.8)	6.3 (4.7–9.4)	0.65
HOMA-IR	21.2	16.9 (4.7–36.7)	22.7 (8.3–45.8)	16.3 (5.3–40.1)	0.67

Data are presented as median (interquartile range). * Kruskal–Wallis test with post hoc Conover test was used. † Statistically significant difference (*p* < 0.05) observed between moderate and severe groups: (2) vs. (3).

**Table 4:** Descriptive statistics of measured and glucose-corrected sodium concentrations.

	Number (%) of Patients	Median (IQR)	Range
Sodium	252	138 (135–141)	111–166
Corrected sodium	252	139 (136–142)	111–166.4

Serum sodium was corrected for hyperglycaemia using a correction factor of 1.6 mmol/L for glucose concentrations between 5.6 and 22 mmol/L and 2.4 mmol/L for glucose concentrations >22 mmol/L.

**Table 5:** Distribution of patients according to sodium status before and after glucose correction.

Sodium Status	Number (%) of Patients		
	Before Correction	After Correction	Absolute Change
Hyponatremia	53 (21.0)	42 (16.7)	−11
Normonatremia	183 (72.6)	187 (74.2)	+4
Hypernatremia	16 (6.3)	23 (9.1)	+7

Serum sodium was corrected for hyperglycaemia using a correction factor of 1.6 mmol/L for glucose concentrations between 5.6 and 22 mmol/L and 2.4 mmol/L for glucose concentrations >22 mmol/L.

**Table 6:** Measured and corrected sodium concentrations according to disease severity.

All Patients	Median (Interquartile Range) According to Disease Severity			*p* *
	Moderate Disease (n = 45)	Severe Disease (n = 158)	Critical Disease (n = 48)	
	(2)	(3)	(4)	
Measured sodium	139 (135–142)	138 (135–141)	138 (135–141)	0.38
Corrected sodium	140 (137–143)	139 (136–141)	138 (136–141)	0.32

* Kruskal–Wallis test. The patient with mild disease (n = 1) was excluded from the analysis because the group contained only one observation.

**Table 7:** Distribution of patients according to the degree of hyponatremia and hypernatremia.

	Number (%) of Patients
Hyponatremia	[42 (16.7)]
Mild	34 (13.5)
Moderate	6 (2.4)
Severe	2 (0.8)
Hypernatremia	[23 (9.1)]
Mild	14 (5.6)
Moderate	6 (2.4)
Severe	3 (1.2)

**Table 8:** Distribution of patients according to disease severity by the degree of hyponatremia.

	Number (%) of Patients with Hyponatremia				*p* *
	Mild (n = 34)	Moderate (n = 6)	Severe (n = 2)	Total (n = 42)	
Disease severity					
Moderate disease	5 (15)	2 (33)	0	7 (16.7)	0.78
Severe disease	24 (71)	3 (50)	2 (100)	29 (69.0)	
Critical disease	5 (15)	1 (17)	0	6 (14.3)	

* Fisher’s exact test.

**Table 9:** Distribution of patients according to ICU admission and clinical outcome by the degree of hyponatremia.

	Number (%) of Patients with Hyponatremia				*p* *
	Mild (n = 34)	Moderate (n = 6)	Severe (n = 2)	Total (n = 42)	
ICU admission					
No	26 (76)	5 (83)	2 (100)	33 (79)	>0.99
Yes	8 (24)	1 (17)	0	9 (21)	
Clinical outcome					
Survived	25 (74)	5 (83)	2 (100)	32 (76)	>0.99
Died	9 (26)	1 (17)	0	10 (24)	

* Fisher’s exact test.

**Table 10:** Distribution of patients according to disease severity by the degree of hypernatremia.

	Number (%) of Patients with Hypernatremia				*p* *
	Mild (n = 14)	Moderate (n = 6)	Severe (n = 3)	Total (n = 23)	
Disease severity					
Moderate disease	5 (36)	0	0	5 (22)	0.34
Severe disease	6 (43)	5 (83)	2 (67)	13 (57)	
Critical disease	3 (21)	1 (17)	1 (33)	5 (22)	

* Fisher’s exact test.

**Table 11:** Distribution of patients according to ICU admission and clinical outcome by the degree of hypernatremia.

	Number (%) of Patients with Hypernatremia				*p* *
	Mild (n = 14)	Moderate (n = 6)	Severe (n = 3)	Total (n = 23)	
ICU admission					
No	13 (93)	4 (67)	2 (67)	19 (83)	0.18
Yes	1 (7)	2 (33)	1 (33)	4 (17)	
Clinical outcome					
Survived	9 (64)	2 (33)	2 (67)	13 (57)	0.48
Died	5 (36)	4 (67)	1 (33)	10 (43)	

* Fisher’s exact test.

**Table 12:** Sodium concentrations according to ICU admission.

Sodium	Median (IQR)		† Difference	95% Confidence Interval	*p* *
	ICU-No	ICU-Yes			
All patients (n = 201:51)	139 (136–141)	139 (136–142)	0.04	−1.21 to 1.61	0.89
Female (n = 103:24)	139 (136–142)	139 (136–144)	0.18	−2.22 to 2.63	0.88
Male (n = 98:27)	138 (136–141)	138 (136–141)	0.17	−1.63 to 2.06	0.78

IQR—interquartile range; * Mann–Whitney U test; † Hodges–Lehmann estimate of the median difference.

**Table 13:** Sodium concentrations according to clinical outcome.

Sodium	Median (IQR)		† Difference	95% Confidence Interval	*p* *
	Survived	Deceased			
All patients (n = 184:68)	139 (136–141)	140 (137–143)	1.37	0 to 2.87	**0.04**
Female (n = 88:39)	139 (136–142)	140 (137–145)	1.36	−0.77 to 3.49	0.19
Male (n = 96:29)	138 (135–141)	140 (136–142)	1.37	−0.29 to 3.16	0.13

IQR—interquartile range; * Mann–Whitney U test; † Hodges–Lehmann estimate of the median difference. Bold values indicate statistically significant results.

**Table 14:** Hormonal and metabolic parameters according to ICU admission.

	Median (IQR)		† Difference	95% Confidence Interval	*p* *
	ICU-No	ICU-Yes			
All patients	n = 201	n = 51			
Sodium	139 (136–141)	139 (136–142)	0.04	−1.21 to 1.61	0.89
TSH [mIU/L]	0.9 (0.5–1.6)	0.8 (0.3–1.7)	−0.06	−0.28 to 0.17	0.56
FT3 [pmol/L]	3.1 (2.6–3.8)	3 (2.4–3.7)	−0.08	−0.36 to 0.21	0.58
FT4 [pmol/L]	17.8 (15.2–20.4)	17 (14.5–19.8)	−0.9	−2.1 to 0.5	0.23
Cortisol [nmol/L]	589.5 (389.8–918)	668 (420.5–889.5)	23	−108 to 141	0.71
Vitamin D3 [nmol/L]	28.2 (15.7–41.3)	25.6 (15.4–40.4)	0	−5.3 to 5.0	0.99
Glucose [mmol/L]	6 (4.7–8.6)	7 (5.3–10.7)	0.97	0.04 to 1.9	**0.04**
HOMA-IR	17.4 (5.9–39)	29.4 (11.7–75.1)	10.1	0.8 to 21.8	**0.02**
Female	n = 103	n = 24			
Sodium	139 (136- 142)	139 (136–144)	0.18	−2.22 to 2.63	0.88
TSH [mIU/L]	1.1 (0.5–2.1)	0.7 (0.3–1.7)	−0.23	−0.65 to 0.12	0.16
FT3 [pmol/L]	3.1 (2.6–3.8)	2.7 (2.3–3.5)	−0.31	−0.65 to 3.43	0.09
FT4 [pmol/L]	18.3 (15.9–22.1)	16.5 (14.6–19.2)	−1.8	−3.6 to −0.1	**0.04**
Cortisol [nmol/L]	609 (383–978)	671.5 (369.3–931)	−9	−217 to 186	0.96
Vitamin D3 [nmol/L]	24.9 (13.8–39.2)	19.8 (14.9–34.5)	−2	−9.2 to 4.4	0.62
Glucose [mmol/L]	6.2 (4.5–8.7)	7.6 (5.6–12.4)	1.45	0.15 to 2.93	**0.03**
HOMA-IR	16.2 (6.3–37.2)	29.6 (7.4–67.8)	8.2	−4.18 to 29.6	0.21
The bold fo	n = 98	n = 27			
Sodium	138 (136–141)	138 (136–141)	0.17	−1.63 to 2.06	0.78
TSH [mIU/L]	0.8 (0.5–1.3)	1 (0.5–1.8)	0.09	−0.18 to 0.42	0.52
FT3 [pmol/L]	3.1 (2.6–4)	3.3 (2.8–3.9)	0.13	−029 to 0.56	0.53
FT4 [pmol/L]	16.7 (14.6–19.2)	17.1 (14.5–19.9)	0.3	−1.8 to 2.2	0.73
Cortisol [nmol/L]	538 (392–847)	610 (418–758)	49	−113 to 191	0.47
Vitamin D3 [nmol/L]	29.5 (19.2–45.8)	33 (21.5–47.4)	1.4	−6.2 to 9.3	0.73
Glucose [mmol/L]	6 (4.9–8.4)	6.9 (5.3–9.9)	0.54	−0.78 to 1.67	0.41
HOMA-IR	22.5 (5.5–39.5)	29.3 (14.2–98.4)	10.4	−2.71 to 27.7	0.11

* Mann–Whitney U test; † Hodges–Lehmann estimate of the median difference. Bold values indicate statistically significant results. Data are presented as median (interquartile range).

**Table 15:** Hormonal and metabolic parameters according to survival status.

	Median (IQR)		† Difference	95% Confidence Interval	*p* *
	Survived	Deceased			
All patients	n = 184	n = 68			
Sodium	139 (136–141)	140 (137–143)	1.37	0 to 2.87	**0.04**
FT4 [pmol/L]	17.7 (15.1–20.6)	17 (14.9–19.1)	−0.8	−1.9 to 0.4	0.19
FT3 [pmol/L]	3.2 (2.7–4)	2.8 (2.1–3.5)	−0.5	−0.7 to −0.2	**<0.001**
Cortisol [nmol/L]	555 (376–895)	684.5 (495–1015.8)	136	24 to 247	**0.02**
Vitamin D3 [nmol/L]	29.7 (17.5–42.9)	21.5 (12.9–34.2)	−6.7	−11.7 to −2.1	**0.003**
Glucose [mmol/L]	6 (4.7–8.5)	7 (5.3–10.1)	0.8	−0.003 to 1.6	0.06
HOMA-IR	20.5 (8–41.6)	21.9 (6–51.3)	−0.12	−7.5 to 6.8	0.91
Female	n = 88	n = 39			
Sodium	139 (136–142)	140 (137–145)	1.36	−0.77 to 3.49	0.19
TSH [mIU/L]	1.2 (0.5–2.2)	0.7 (0.3–1.6)	−0.4	−0.8 to −0.04	**0.03**
FT3 [pmol/L]	3.1 (2.6–3.9)	2.7 (2.1–3.1)	−0.5	−0.8 to −0.2	**0.002**
Cortisol [nmol/L]	590 (359.5–907)	745.5 (522.8–1033.8)	153.5	−13 to 324	0.07
Vitamin D3 [nmol/L]	28.7 (17.1–40)	15.6 (11.8–25.2)	−8.1	−14.6 to −2.2	**0.008**
Glucose [mmol/L]	6.3 (4.5–8.9)	7 (4.8–9.5)	0.42	−0.59 to 1.6	0.37
HOMA-IR	16.9 (7.7–45.5)	16.2 (6.3–45)	−1.5	−9.9 to 7.5	0.69
Male	n = 96	n = 29			
Sodium	138 (135–141)	140 (136–142)	1.37	−0.29 to 3.16	0.13
TSH [mIU/L]	0.8 (0.5–1.3)	0.8 (0.5–1.8)	0.02	−0.26 to 0.33	0.92
FT3 [pmol/L]	3.3 (2.8–4)	3 (2.1–3.7)	−0.39	−0.8 to 0.06	0.08
Cortisol [nmol/L]	537 (388.3–816.3)	647.5 (457.3–897)	103	−51 to 263	0.16
Vitamin D3 [nmol/L]	32.3 (19.9–47.5)	27.6 (19.7–37.6)	−4.1	−12 to 3	0.22
Glucose [mmol/L]	5.9 (4.8–8)	6.9 (5.6–10.4)	1.1	0 to 2.3	0.05
HOMA-IR	23.6 (8.2–39.9)	23.9 (5.6–58.9)	1.3	−9.6 to 16.3	0.75

* Mann–Whitney U test; † Hodges–Lehmann estimate of the median difference. Bold values indicate statistically significant results. Data are presented as median (interquartile range).

## Data Availability

All data generated or analyzed throughout this study are included within the article. Additional data that supports the findings of this research may be made available by the corresponding author on request, provided that ethical, legal, and privacy considerations are adhered to. This research constitutes a segment of the first author’s doctoral dissertation, with plans for further analyses of the dataset to be published in future studies.

## Abbreviations

The following abbreviations are used in this manuscript:

ACE2	Angiotensin-converting enzyme 2
ALP	Alkaline phosphatase
ALT	Alanine aminotransferase
AST	Aspartate aminotransferase
AUC	Area under the curve
CI	Confidence interval
CK	Creatine kinase
Cl	Chloride
COVID-19	Coronavirus disease 2019
CRE	Creatinine
CRP	C-reactive protein
FT3	Free triiodothyronine
FT4	Free thyroxine
GGT	Gamma-glutamyl transferase
GH	Human growth hormone
HFNC	High-flow nasal cannula
HOMA-IR	Homeostasis model assessment of insulin resistance
ICU	Intensive care unit
IGF-1	Insulin-like growth factor 1
IQR	Interquartile range
K	Potassium
LOS	Length of stay
mL	milliliter
L	liter
n	Sample size
Na	Sodium
ng	nanogram
OR	Odds ratio
*p* value	*p*-value
pg	picogram
RDW	Red cell distribution width
SARS-CoV-2	Severe acute respiratory syndrome coronavirus 2
TSH	Thyroid-stimulating hormone
YI	Youden index

## Appendix A

**Table A1:** Distribution of patients according to ICU admission.

	Median (IQR)		† Difference	95% Confidence Interval	*p* *
	ICU-No	ICU-Yes			
All patients	n = 201	n = 51			
Sodium	139 (136–141)	139 (136–142)	0.04	−1.21 to 1.61	0.89
TSH [mIU/L]	0.9 (0.5–1.6)	0.8 (0.3–1.7)	−0.06	−0.28 to 0.17	0.56
FT4 [pmol/L]	17.8 (15.2–20.4)	17 (14.5–19.8)	−0.9	−2.1 to 0.5	0.23
FT3 [pmol/L]	3.1 (2.6–3.8)	3 (2.4–3.7)	−0.08	−0.36 to 0.21	0.58
Cortisol [nmol/L]	589.5 (389.8–918)	668 (420.5–889.5)	23	−108 to 141	0.71
GH [ng/mL]	0.6 (0.3–1.3)	0.3 (0.2–1)	−0.16	−0.37 to 0.01	0.06
IGF-1 [nmol/L]	7.7 (4.8–12.2)	9.1 (4.6–11.5)	0.40	−1.6 to 2.3	0.70
Insulin [pmol/L]	71.9 (36.7–130)	83.5 (43.7–158.8)	9.2	−14.7 to 35.5	0.41
Vitamin D3 [nmol/L]	28.2 (15.7–41.3)	25.6 (15.4–40.4)	0	−5.3 to 5.0	0.99
Glucose [mmol/L]	6 (4.7–8.6)	7 (5.3–10.7)	0.97	0.04 to 1.9	**0.04**
HOMA-IR	17.4 (5.9–39)	29.4 (11.7–75.1)	10.1	0.8 to 21.8	**0.02**
Female	n = 103	n = 24			
Sodium	139 (136–142)	139 (136–144)	0.18	−2.22 to 2.63	0.88
TSH [mIU/L]	1.1 (0.5–2.1)	0.7 (0.3–1.7)	−0.23	−0.65 to 0.12	0.16
FT4 [pmol/L]	18.3 (15.9–22.1)	16.5 (14.6–19.2)	−1.8	−3.6 to −0.1	**0.04**
FT3 [pmol/L]	3.1 (2.6–3.8)	2.7 (2.3–3.5)	−0.31	−0.65 to 3.43	0.09
Cortisol [nmol/L]	609 (383–978)	671.5 (369.3–931)	−9	−217 to 186	0.96
GH [ng/mL]	0.7 (0.3–1.5)	0.4 (0.2–0.7)	−0.26	−0.67 to −0.01	**0.03**
IGF-1 [nmol/L]	7.3 (4.1–10.8)	5.8 (3.5–9.6)	−1.19	−3.79 to 1.25	0.39
Insulin [pmol/L]	70 (36.7–124.5)	113 (26.7–183)	15.4	−22.1 to 66.6	0.39
Vitamin D3 [nmol/L]	24.9 (13.8–39.2)	19.8 (14.9–34.5)	−2	−9.2 to 4.4	0.62
Glucose [mmol/L]	6.2 (4.5–8.7)	7.6 (5.6–12.4)	1.45	0.15 to 2.93	**0.03**
HOMA-IR	16.2 (6.3–37.2)	29.6 (7.4–67.8)	8.2	−4.18 to 29.6	0.21
Male	n = 98	n = 27			
Sodium	138 (136–141)	138 (136–141)	0.17	−1.63 to 2.06	0.78
TSH [mIU/L]	0.8 (0.5–1.3)	1 (0.5–1.8)	0.09	−0.18 to 0.42	0.52
FT4 [pmol/L]	16.7 (14.6–19.2)	17.1 (14.5–19.9)	0.3	−1.8 to 2.2	0.73
FT3 [pmol/L]	3.1 (2.6–4)	3.3 (2.8–3.9)	0.13	−0.29 to 0.56	0.53
Cortisol [nmol/L]	538 (392–847)	610 (418–758)	49	−113 to 191	0.47
GH [ng/mL]	0.5 (0.3–1.1)	0.3 (0.2–1.3)	−0.09	−0.34 to 0.13	0.53
IGF-1 [nmol/L]	8.2 (5.4–12.7)	10.2 (8.5–12.2)	1.54	−1.3 to 4.3	0.32
Insulin [pmol/L]	89.9 (34.5–131)	61.8 (44.6–157.5)	3.7	−30 to 35.3	0.82
Vitamin D3 [nmol/L]	29.5 (19.2–45.8)	33 (21.5–47.4)	1.4	−6.2 to 9.3	0.73
Glucose [mmol/L]	6 (4.9–8.4)	6.9 (5.3–9.9)	0.54	−0.78 to 1.67	0.41
HOMA-IR	22.5 (5.5–39.5)	29.3 (14.2–98.4)	10.4	−2.71 to 27.7	0.11

* Mann-Whitney U test; † Hodges–Lehmann estimate of the median difference. Bold values indicate statistically significant results. Data are presented as median (interquartile range).

**Table A2:** Distribution of patients according to outcome across groups.

	Median (IQR)		† Difference	95% Confidence Interval	*p* *
	Survived	Deceased			
All patients	n = 184	n = 68			
Sodium	139 (136−141)	140 (137−143)	1.37	0 to 2.87	**0.04**
TSH [mIU/L]	0.9 (0.5–1.7)	0.7 (0.4–1.6)	−0.14	−0.35 to 0.05	0.13
FT4 [pmol/L]	17.7 (15.1–20.6)	17 (14.9–19.1)	−0.8	−1.9 to 0.4	0.19
FT3 [pmol/L]	3.2 (2.7−4)	2.8 (2.1–3.5)	−0.5	−0.7 to −0.2	**<0.001**
Cortisol [nmol/L]	555 (376−895)	684.5 (495–1015.8)	136	24 to 247	**0.02**
GH [ng/mL]	0.5 (0.2–1.2)	0.6 (0.3–1.4)	0.04	−0.13 to 0.21	0.59
IGF-1 [nmol/L]	7.7 (5–11.8)	8.7 (3.9–11.6)	−0.20	−1.94 to 1.16	0.82
Insulin [pmol/L]	72 (39.5–132.5)	76.4 (21.4−135)	−8	−28.7 to 15.0	0.49
Vitamin D3 [nmol/L]	29,7 (17.5–42.9)	21,5 (12.9−34.2)	−6.7	−11.7 to −2.1	**0.003**
Glucose [mmol/L]	6 (4.7–8.5)	7 (5.3–10.1)	0.8	−0.03 to 1.6	0.06
HOMA-IR	20.5 (8–41.6)	21.9 (6–51.3)	−0.12	−7.5 to 6.8	0.91
Female	n = 88	n = 39			
Sodium	139 (136−142)	140 (137−145)	1.36	−0.77 to 3.49	0.19
TSH [mIU/L]	1.2 (0.5–2.2)	0.7 (0.3–1.6)	−0.4	−0.8 to −0.04	**0.03**
FT4 [pmol/L]	18.3 (15.7–22.2)	17.6 (15–19.3)	−1.1	−2.7 to 0.4	0.17
FT3 [pmol/L]	3.1 (2.6–3.9)	2.7 (2.1–3.1)	−0.5	−0.8 to −0.2	**0.002**
Cortisol [nmol/L]	590 (359.5−907)	745.5 (522.8–1033.8)	153.5	−13 to 324	0.07
GH [ng/mL]	0.7 (0.3–1.4)	0.4 (0.3–1.1)	−0.08	−0.43 to 0.13	0.49
IGF-1 [nmol/L]	7.3 (4.3–10.4)	6.3 (3.4–9.9)	−0.24	−2.6 to 1.9	0.83
Insulin [pmol/L]	71.2 (39.1–142.3)	76.4 (17.3−128)	−8	−36 to 26	0.64
Vitamin D3 [nmol/L]	28.7 (17.1−40)	15.6 (11.8–25.2)	−8.1	−14.6 to −2.2	**0.008**
Glucose [mmol/L]	6.3 (4.5–8.9)	7 (4.8–9.5)	0.42	−0.59 to 1.6	0.37
HOMA-IR	16.9 (7.7–45.5)	16.2 (6.3−45)	−1.5	−9.9 to 7.5	0.69
Male	n = 96	n = 29			
Sodium	138 (135−141)	140 (136−142)	1.37	−0.29 to 3.16	0.13
TSH [mIU/L]	0.8 (0.5–1.3)	0.8 (0.5–1.8)	0.02	−0.26 to 0.33	0.92
FT4 [pmol/L]	16.9 (14.7–19.7)	16.9 (13.6−19)	−0.70	−2.7 to 1.0	0.44
FT3 [pmol/L]	3.3 (2.8−4)	3 (2.1–3.7)	−0.39	−0.8 to 0.06	0.08
Cortisol [nmol/L]	537 (388.3–816.3)	647.5 (457.3−897)	103	−51 to 263	0.16
GH [ng/mL]	0.4 (0.2–1.1)	0.9 (0.2–1.6)	0.17	−0.09 to 0.60	0.17
IGF-1 [nmol/L]	9.7 (5.5–12.4)	9.7 (5.4–12.8)	0.23	−2.37 to 3.1	0.87
Insulin [pmol/L]	90.3 (39.5–132.5)	74.7 (24.7−150)	−6.6	−39.1 to 28.6	0.66
Vitamin D3 [nmol/L]	32.3 (19.9–47.5)	27.6 (19.7–37.6)	−4.1	−12 to 3	0.22
Glucose [mmol/L]	5.9 (4.8−8)	6.9 (5.6–10.4)	1.1	0 to 2.3	0.05
HOMA-IR	23.6 (8.2–39.9)	23.9 (5.6–58.9)	1.3	−9.6 to 16.3	0.75

* Mann-Whitney U test; † Hodges–Lehmann estimate of the median difference. Bold values indicate statistically significant results. Data are presented as median (interquartile range).

**Table A3:** Multivariate logistic regression models for severe and critical disease, ICU admission, and mortality.

Predictor	Regression Coefficient ß	*p* Value	OR	95% Confidence Interval
Severe and critical disease				
Age	−0.007	0.59	0.99	0.97 to 1.02
Sex	−0.251	0.49	0.78	0.38 to 1.58
Arterial hypertension	−0.257	0.60	0.77	0.29 to 2.00
Type 2 diabetes mellitus	0.199	0.61	1.22	0.56 to 2.64
Glucose	−0.026	0.35	0.97	0.92 to 1.03
CRP	0.001	0.81	1.00	0.99 to 1.01
Dysnatremia and/or endocrine alterations	0.250	0.49	1.28	0.632 to 2.61
ICU admission				
Age	−0.005	0.71	1.00	0.97 to 1.02
Sex	0.065	0.85	1.07	0.53 to 2.14
Arterial hypertension	−0.471	0.27	0.62	0.27 to 1.44
Type 2 diabetes mellitus	−0.489	0.23	0.61	0.28 to 1.36
Glucose	0.064	**0.02**	1.07	1.01 to 1.13
CRP	0.004	0.05	1.00	1.00 to 1.01
Dysnatremia and/or endocrine alterations	0.118	0.74	1.13	0.55 to 2.29
Mortality				
Age	0.022	0.08	1.02	0.99 to 1.05
Sex	−0.301	0.34	0.74	0.39 to 1.38
Arterial hypertension	−0.239	0.54	0.79	0.36 to 1.71
Type 2 diabetes mellitus	0.246	0.47	1.28	0.65 to 2.52
Glucose	−0.016	0.59	0.98	0.93 to 1.04
CRP	0.004	0.05	1.00	1.00 to 1.01
Dysnatremia and/or endocrine alterations	0.321	0.32	1.38	0.73 to 2.59

OR—Odds ratio; Bold values indicate statistically significant results.
